# Id4 deficiency attenuates prostate development and promotes PIN-like lesions by regulating androgen receptor activity and expression of NKX3.1 and PTEN

**DOI:** 10.1186/1476-4598-12-67

**Published:** 2013-06-21

**Authors:** Pankaj Sharma, Ashley Evans Knowell, Swathi Chinaranagari, Shravan Komaragiri, Peri Nagappan, Divya Patel, Mathew C Havrda, Jaideep Chaudhary

**Affiliations:** 1 Center for Cancer Research and Therapeutics Development, Clark Atlanta University, Atlanta, GA, USA; 2 Norris Cotton Cancer Center, Lebanon, NH, USA; 3 Geisel Medical School at Dartmouth, Lebanon, NH, USA; 4 Center for Cancer Research and Therapeutics Development, Department of Biological Sciences, Clark Atlanta University, 223 James P. Brawley Dr. SW, Atlanta, GA 30314, USA

**Keywords:** Id4, Prostate, Androgen receptor, NKX3.1, Akt, PTEN

## Abstract

**Background:**

Inhibitor of differentiation 4 (Id4), a member of the helix-loop-helix family of transcriptional regulators has emerged as a tumor suppressor in prostate cancer. Id4 is expressed in the normal prostate where its expression is also regulated by androgens. In this study we investigated the effect of loss of Id4 (Id4-/-) on adult prostate morphology.

**Methods:**

Histological analysis was performed on prostates from 6-8 weeks old Id4-/-, Id4+/- and Id4+/+ mice. Expression of Id1, Sox9, Myc, androgen receptor, Akt, p-Akt, Pten and Nkx3.1 was investigated by immunohistochemistry. Androgen receptor binding on NKX3.1 promoter was studied by chromatin immuno-precipitation. Id4 was either over-expressed or silenced in prostate cancer cell lines DU145 and LNCaP respectively followed by analysis of PTEN, NKX3.1 and Sox9 expression.

**Results:**

Id4-/- mice had smaller prostates with fewer tubules, smaller tubule diameters and subtle mPIN like lesions. Levels of androgen receptor were similar between wild type and Id4-/- prostate. Decreased NKX3.1 expression was in part due to decreased androgen receptor binding on NKX3.1 promoter in Id4-/- mice. The increase in the expression of Myc, Sox9, Id1, Ki67 and decrease in the expression of PTEN, Akt and phospho-AKT was associated with subtle mPIN like lesions in Id4-/- prostates. Finally, prostate cancer cell line models in which Id4 was either silenced or over-expressed confirmed that Id4 regulates NKX3.1, Sox9 and PTEN.

**Conclusions:**

Our results suggest that loss of Id4 attenuates normal prostate development and promotes hyperplasia/dysplasia with subtle mPIN like lesions characterized by gain of Myc and Id1 and loss of Nkx3.1 and Pten expression. One of the mechanisms by which Id4 may regulate normal prostate development is through regulating androgen receptor binding to respective response elements such as those on NKX3.1 promoter. In spite of these complex alterations, large neoplastic lesions in Id4-/- prostates were not observed suggesting the possibility of mechanisms/pathways such as loss of Akt that could restrain the formation of significant pre-cancerous lesions.

## Background

Id4 (inhibitor of differentiation-4), is a member of the inhibitor of differentiation (Id) gene family (Id1, Id2 and Id3) and acts as a transcriptional regulator of basic helix-loop-helix (bHLH) family of transcription factors [1]. Due to lack of the basic DNA binding domain, Id4 (and all Id proteins) acts as a dominant negative regulator of bHLH transcription factors, most notably E2A (TCF3) [1,2].

The interaction repertoire of Id proteins also involves several non-bHLH proteins. Whereas all Id proteins interact with bHLH TCF3, their interaction with non-bHLH proteins appears in large part to be isoform dependent- Id1: CASK, ELK1, GATA4, caveolin; Id2: ELK1, 3 and 4, CDK2, PAX2, 5 and 8, Rb and related pocket proteins, Id3: ELK1 and 4, ADD1 ([1,2] and public databases). Specific non-bHLH interaction partners for Id4 are currently not known. Thus Id proteins are capable of regulating the expression of a large number of genes through specific bHLH and non-bHLH interactions that in turn regulates many cellular processes such as cell growth, differentiation, and apoptosis [3].

Id proteins are expressed by essentially all cell lineages at some point of development. In general, Id expression is highest in undifferentiated, proliferating populations and is down-regulated as cells exit from cell cycle and terminally differentiate (reviewed in [1-3]). Knock out mouse models evaluating Id genes have demonstrated their essential role in development. Id2 null mice displays phenotypic abnormalities of retarded growth and neonatal morbidity due to a lactation defect [4], impaired chondrogenesis [5], B cell development [6] and severe cardiac defects [7]. Male Id2-/- mice also exhibit defects in spermatogenesis [8]. Id3 null mice develops primary Sjögren’s syndrome-like symptoms [9], specific defects in B/T lymphocyte development [10], and restricted development of the gamma delta lineage during thymopoiesis [11]. Interestingly, no phenotype is observed in mice lacking only Id1 suggesting that its function can be effectively compensated by the other three Ids. So far embryonic lethality has been observed only in mice homozygously lacking both Id1 and Id3 suggesting that Id1 and Id3 may have many overlapping functions [12]. Id4 is required for normal brain size and lateral expansion of the proliferative zone in the developing cortex and hippocampus possibly by regulating neural stem cell proliferation and differentiation [13]. Id4 is also required for normal mammary gland development in p38MAPK dependent pathway [14] and for spermatogonial stem cell renewal [15].

Studies have also shown that unlike other Ids, Id4 promotes differentiation in many systems including osteoblast [16], adipocytes [17], neurons [13] and oligodendrocytes [18]. Paradoxically, Id4 appears to demonstrate both pro-tumor and anti-tumor properties. Epigenetic silencing of Id4 in leukemia [19], breast [20,21], colorectal [22] mouse and human CLL (chronic lymphocytic leukemia [23]) and gastric cancer [24] tend to support its anti-tumor activity. Whereas high Id4 expression is reported in B-cell acute lymphoblastic leukemia [25] and B-cell precursor acute lymphoblastic leukemia (BCP-ALL) [26] due to the t(6;14)(p22;q32) chromosomal translocation, and in bladder [27] and rat mammary gland carcinomas [28] suggests that it may also have pro-tumor activity.

We and others have recently shown that Id4 is highly expressed in the normal prostate and decreased in prostate cancer due to promoter hypermethylation [29,30]. Id4 expression in the prostate thus appears in contrast with the expression of other Id genes (Id1 and Id3) which are expressed at low to negligible levels in the normal prostate although their expression increases significantly in prostate cancer [31-33]. Moreover, Id4 is regulated by androgens in cells that respond to androgen stimulation such as testicular Sertoli cells and prostate epithelial cells [34]. Id4 also restores androgen receptor expression and activity in the androgen receptor negative prostate cancer cell line DU145 [35]. These results suggest that Id4 could potentially act within the androgen receptor pathway to regulate the development and function of the prostate. We used the Id4 -/- mouse model to evaluate further the role of Id4 in prostate development and its significance in prostate cancer. Our findings suggest that Id4 is required for normal prostate development. The prostate in Id4-/- mice have a complex phenotype characterized by attenuated growth and development that also mimics subtle features of prostatic intraepithelial neoplasia (PIN).

## Results

### Id4 is expressed in the normal mouse prostate

**Figure 1:**
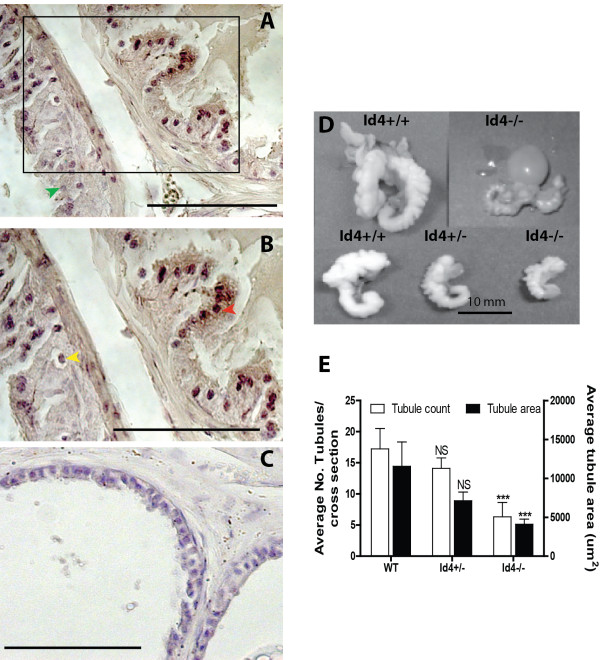
**Effect of loss of Id4 on mouse prostate development. A** and **B** – Immuno-histochemical analysis of Id4 expression in the wild type mouse prostate. Panel **B** is the enlarged version of the box represented in Panel **A**. Id4 expression is primarily localized to epithelial cell nuclei. Red, green and yellow arrow heads represent cells expressing high, low or no Id4 respectively. **C**: Lack of Id4 expression in Id4-/- prostates. The scale bars are 100um. **D**: The relative size of the genital tract (excluding testis and epididymis) of the wild type (Id4+/+) and mutant (Id4-/-) mice. The representative image of three different tracts demonstrating clear size differences is shown. The bottom panel represents the size of the seminal vesicle from the Id4+/+, Id4+/- and Id4-/- mice. **E**: Average number of tubules and tubule diameter per cross section in the wild type and Id4 -/- prostates. The number of tubules were counted in each section (n = 100 serial cross sections for Id4+/+, n = 76 for Id4+/- and n = 54 for Id4-/, proximal to distal) at 50× and their mean ± SEM is indicated as open bars. The black bars represent average tubule diameters (±SEM) of the number of tubules counted in each of the serial sections at 50×. (*** P < 0.001, NS: non-significant).

In this study we demonstrate that Id4 is highly expressed in the adult mouse prostate glandular epithelial cells (Figure 1A and B) with little to no expression in the adjacent stroma. While the majority of glandular epithelial cells stained strongly positive for Id4 (red arrow, Figure 1B), the staining intensity in few cells was lower (yellow arrow, Figure 1B) or absent (green arrow, Figure 1A). These low to negative Id4 cells were found interspersed suggesting cell-cell variability in Id4 expression. Id4 expression in the mouse prostate is therefore similar to human prostate in which Id4 expression is readily observed in most of the epithelial cells. We then used the Id4-/- mice prostates (Figure 1C) to investigate its role in prostate development.

### Severe genital tract (GT) phenotype in male Id4-/- mice

The genital tract (GT) size of Id4-/- mice was noticeably smaller as compared to the wild type mice (Figure 1D). The GT size of heterozygous mice (Id4+/-) was intermediate between Id4+/+ and Id4-/- mice. The prostates and seminal vesicles (Figure 1D bottom panel) were also visibly smaller in Id4-/- mice suggesting that Id4 is required for normal genital tract development. Previous studies using the same Id-/- model have shown similar levels of circulating testosterone between Id4+/+ and Id4-/- mice [15]. These results suggested that the smaller genital tract in Id4-/- mice was not due to lower testosterone levels.

### Loss of Id4 results in impaired prostate development

**Figure 2:**
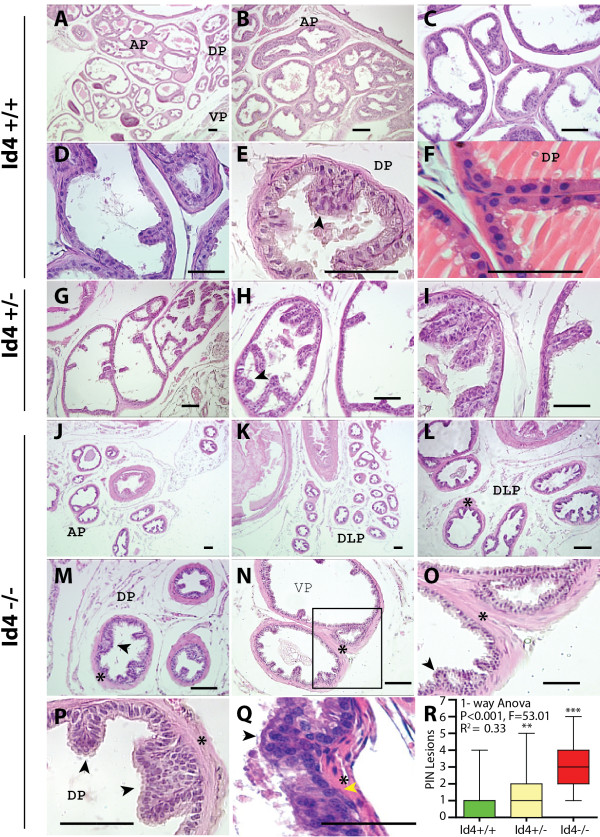
**Hematoxylin and Eosin staining of the wild type (Id4+/+, A-F), heterozygous (Id4+/-, G-I) and homozygous (Id4-/-, J-Q) mouse prostates (5 um sections from proximal to distal region).** The yellow (Panel **Q**) and black arrowheads (Panels **E**, **M**, **O**, **P**, **Q**) represent hyperchromatic nuclei and layers of stratified epithelium respectively. The asterisk indicates the stromal layer surrounding the tubules (Panels **O**, **P** and **Q**). Panel **O** is an enlarged image of the corresponding box in panel **N**. Frequency of PIN like lesions (pseudo-stratification) in Id4+/+, Id4+/- and Id4-/- was quantified (average n = 100 cross sections, and >200 tubules) in each genotype and statistical analysis (1-way ANOVA and Dunnett’s multiple comparison test) is shown in panel **R**. Representative images are shown. AP: Anterior prostate, VP: ventral prostate, DLP: Dorso-Lateral Prostate. The sections are counterstained with hematoxylin hence the nuclei are blue. Representative images are shown. The scale bar is 100 um.

Histological analysis indicated a significant decrease between the number and size of prostatic ducts in prostates from Id4-/- mice as compared to age matched littermates. The number of tubules were counted in each section (n = 100 serial cross sections for Id4+/+, n = 76 for Id4+/- and n = 54 for Id4-/-, proximal to distal) at 50x. The average number of tubules and tubule diameter in all the lobes decreased more than three fold in Id4-/- mice (Figure 1E, P < 0.001). Based on glandular histology, all lobes (dorsal, lateral anterior and ventral) were identifiable in the wild type (Figure 2A and B, distal) and Id4-/- (Figure 2J-N, distal, N is more proximal) prostates. The finger like projections typical of anterior prostate appeared to be normal in Id4-/- mice (Figure 2P). Apart from the smaller average diameter (Figure 1E), we routinely saw less eosinophilic serous secretory material within the lumens in Id4-/- prostatic ducts (Figure 2P) as compared to Id4+/+ (Figure 2F).

### Histological analysis of Id4-/- mouse prostates

**Figure 3:**
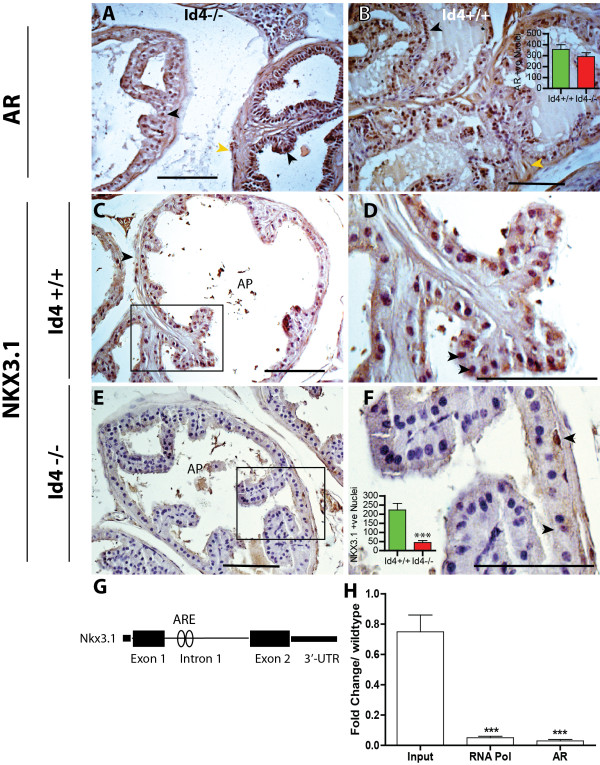
**Loss of Id4 has no effect of Androgen receptor but attenuates NKX3.1 expression.** Androgen Receptor (Panels **A** and **B**) and NKX3.1 expression (Panels **C**-**F**) in wild type (Id4+/+, **B**, **C** and **D**) and Id4 knockout (Id4-/- **A**, **E** and **F**) mice. Androgen receptor (AR) expression was observed in the nucleus (black arrowheads) of both Id4-/- (**A**) and Id4+/+ (**B**) prostate epithelial cells. AR was also observed in the stromal cells (yellow arrowheads) in Id4-/- and Id4+/+ mice. Panels **C** and **D**: Nkx3.1 is highly expressed in the nucleus of wild type prostatic glandular epithelium cells (black arrowheads, Panel **D**). Panel **D** is the enlarged boxed region in Panel **C**. Panel **E**: Nkx3.1 expression is undetectable in mice lacking Id4 (Id4-/-), however at higher magnification (Panel **F**, enlarged boxed region of Panel **E**) some cells stain positive for Nkx3.1 expression (black arrowheads). The brown staining represents the expression of AR in **A** and **B** and NKX3.1 in **C**-**F**. Representative images are shown. The AR and Nkx3.1 positive cells were counted in 25 tubules each in Id4+/+ and Id4-/- cross sections. The average AR or Nkx3.1 positive cells per tubule is shown in Panels **B** and **F** respectively (***: P < 0.001, *t*-test, n = 25 tubules). The scale bar is 100um. **G**: Schematic of Nkx3.1 gene including intron 1. The androgen response element (ARE) in intron 1 binds androgen receptor and regulates androgen dependent expression of Nkx3.1 in mice prostate. **H**: Chromatin immuno-precipitation (ChIP) based analysis of androgen receptor binding to the ARE site in intron 1 of Nkx3.1. The binding of RNA polymerase I (RNA Pol) and AR was quantitated using real time PCR. The data normalized to IgG shows the input, RNA pol and AR in the Id4-/- knockout prostate as compared to wild type set to 1. (*** P < 0.001, n = 3 in triplicate).

In some cross-sections of Id4-/- prostates, extensive layering and pseudo-stratification of the glandular epithelial cells was observed (arrowheads in Figure 2M, O, P and Q). A feature of the Id4-/- lateral prostatic ducts was the presence of abundant fibro muscular stroma surrounding the tubules (asterisk in Figure 2M, N, O, P and Q). The nuclei of Id4-/- mice were hyperchromatic (Yellow arrowhead in Figure 2Q) as compared to the homogenous chromatin found in Id4+/+ nuclei (Figure 2F), suggesting hyperplasia and dysplasia. Some of these abnormalities are consistent with changes associated with prostatic intraepithelial neoplasia (mPIN), which is considered to be a precursor of invasive prostate carcinoma in humans [36]. In fact more mPIN like lesions were observed in Id4-/- prostatic tubules as compared to their wild type counterparts (Figure 2, black arrowheads in panels M, O, P and Q). Quantitation of PIN like lesions (pseudo-stratification) in Id4+/+, Id4+/- and Id4-/- (average n = 100 cross sections, and >200 tubules in each genotype) followed by statistical analysis (1-way ANOVA and Dunnett’s multiple comparison test, Figure 2R) revealed a significant increase in PIN like lesions in Id4-/- mice as compared to Id4+/+ (P < 0.001). Elongated nuclei were also routinely observed in some Id4-/- dorso-lateral and ventral lobe Id4-/- tubules (Figure 2P and Q, see also Figure 3A (arrow)). Presence of elongated and hyperchromatic nuclei are frequently observed in mouse models of prostate cancer such as in LADY (12 T-7f) transgenic mice [37]. This histological analysis revealed that Id4 is required for normal prostate development. Loss of Id4 leads to a decrease in the number of ducts, small tubular size and appearance of subtle PIN like lesions.

### Effect of Id4 on prostate regulatory proteins

We broadly classified the Id4-/- prostate phenotype in two different categories: 1) a hyper-proliferative defect wherein we observed intra-ductal hyperplasia and 2) a developmental defect leading to small prostate size, decreased branching and smaller tubule size. The molecular basis of these alterations was explored by investigating the expression of representative markers associated with each of these two processes.

#### Id4 And prostate development: loss of Id4 has no effect on androgen receptor expression but results in down-regulation of Nkx3.1

Androgen receptor is the key regulator of prostate development including size, branching morphogenesis and differentiation. Quantitation of androgen receptor positive cells (brown nuclei, Figure 3A and B, n = 25 tubules each) followed by statistical analysis revealed that loss of Id4 had no apparent effect on androgen receptor expression (black arrowheads, Figure 3A) as compared to wild type littermates (black arrowheads, Figure 3B and inset) in the glandular epithelium of the prostate. Similar to wild type, AR expression was also present in the stromal cells in Id4-/- prostates (yellow arrowheads, Figure 3A and B). AR was also predominantly nuclear suggesting efficient nuclear translocation in Id4-/- following ligand binding. Thus androgen receptor pathway which is essential to support normal sex differentiation, development of male genital tract and organ development appears to be intact. These results also suggested that Id4 is required to maintain normal prostate development through genetic events downstream of androgen receptor and deficiency of Id4 may attenuate these pathways leading to decreased prostatic secretions and PIN like lesions.

We next investigated the expression of Nkx3.1, a key androgen receptor downstream target. The expression of homeobox gene Nkx3.1 in prostate epithelial cells is rapidly lost after castration, but is quickly restored after androgen dependent prostate regeneration [38]. Nuclear Nkx3.1 expression was clearly observed in prostates from WT mice suggesting a normal prostate developmental program and androgen response (Figure 3C and D). In contrast, Nkx3.1 expression was noticeably absent in the Id4-/- mice (Figure 3E and F and inset, Nkx3.1 positive nuclei counted in n = 25 tubules). Nkx3.1 is also the earliest known marker of prostate development and is a critical regulator of prostate epithelial differentiation in mouse models [39]. Loss of Nkx3.1 leads to significant decreases in prostatic ductal branching and production of secretory proteins [39]. Nkx3.1 knockout mice also frequently display prostate epithelial hyperplasia and dysplasia and often develop PIN [39]. Some of these phenotypes such as reduced ductal branching and diameter (Figure 1D) and PIN like lesions (Figure 2M, O, P, Q and R) were also present in Id4-/- prostates, perhaps due to loss of Nkx3.1 expression.

Androgen-dependent transcription of the mouse Nkx3.1 is conferred through a non-canonical androgen response element (ARE) element within an intron [40] (Figure 3G). Chromatin immuno-precipitation analysis using androgen receptor antibody revealed that AR binding is significantly reduced (P < 0.001) at this site in Id4-/- mice as compared to the levels observed in prostates from WT mice (Figure 3H). These results provided direct evidence that decreased Nkx3.1 expression is not due to loss of androgen receptor (Figure 3A and B) but due to attenuated androgen receptor binding to its cognate response element.

**Figure 4:**
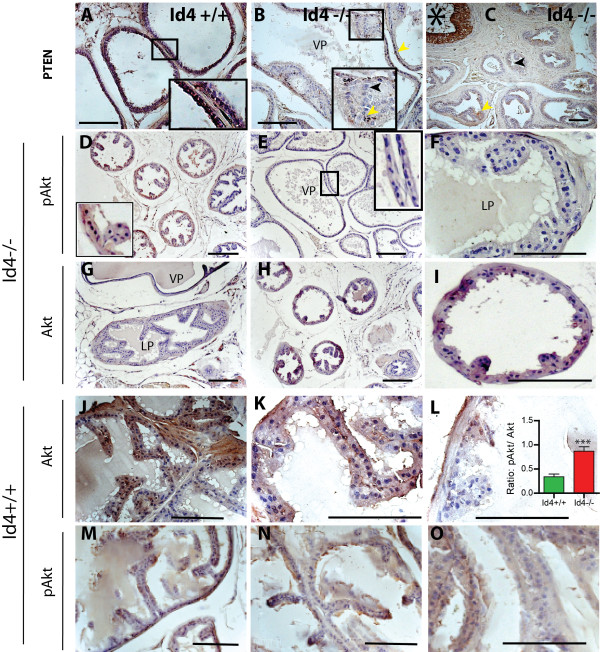
**Pten, Akt and phospho-Akt (p-Akt) expression in wild type (Id4+/+) and Id4 knockout (Id4-/-) mice. A:** Pten was expressed at high level in the normal prostate both in the nucleus and cytoplasm of Id4+/+ prostate. **B** and **C:** Pten expression was significantly reduced or undetectable (black arrowheads) in the Id4-/- prostate ducts. Note the hyperplastic regions in Panel **B**. Occasionally, few Pten positive cells were observed that were primarily localized to epithelial cells near the basement membrane (Inset in Panel **B**). Pten expression was observed in the urethra (asterisk, Panel **C**) but not in prostatic ducts. The inset in Panels **A** and **B** are enlarged boxed regions in corresponding panels. Panels **D**-**F:** Lobe specific expression of phospho-Akt in Id4-/- mice. Increased phospho-Akt was observed in dorsal prostate (Panel **D**) but not in ventral (**E** and inset)) and lateral (**F**) prostate. Phosphorylation of Akt correlated with total Akt expression (**G**-**I**) in Id4-/- prostate. Akt expression was undetectable in lateral and ventral prostate (**G**) but was detectable in dorsal prostate (**H** and **I**) from Id4-/- mice. Panels **J-L:** Total Akt expression in wild type mice prostate. Akt expression was highly variable within the glandular epithelium (**J**). Regions of undetectable to high Akt expression were juxtaposed (**K** and **L**). Similar expression profile (low to high) of phospho-Akt was observed in wild type prostates (Panels **M**-**O**). The cells staining positive for phospho-Akt were counted in tubules that also stained positive for Akt. The ratio of pAkt/Akt positive cells is shown in Panel **L** (***: P < 0.001). Representative images are shown. The scale bar is 100 um.

Based on *in vitro* and *in vivo* studies, PTEN and its downstream signaling pathways have emerged as major regulators of NKX3.1 expression [41]. As expected, Pten was highly expressed in the wild type prostate epithelium and stroma (Figure 4A). The immuno-histochemical studies shown in Figure 4B and C clearly demonstrated a significant decrease in Pten expression in Id4-/- prostate epithelial cells (black arrowheads). Surprisingly, Pten expression was maintained in non-prostatic tissue such as urethra (Figure 4C, asterisk) in Id4-/- mice suggesting that the decreased Pten expression was specific to prostate. Lack of Id4 expression in the urethra (data not shown) further suggests that Pten expression is influenced by Id4 specifically in the prostate. Since Pten regulates Nkx3.1 expression, the loss of prostatic Pten might be an alternate mechanism by which Nkx3.1 is down-regulated in the Id4-/- prostate [42]. Furthermore, these mechanisms may be independent of AR-regulated Nkx3.1 gene transcription mechanism. The Id4-/- knockout model thus closely mimics the Pten:Nkx3.1 mutant mice [43].

Pten, a phosphatase is involved in the regulation of Akt phosphorylation. We measured the expression of phospho-Akt (p-Akt1, 2 and 3) as readout of Pten expression/activity in Id4-/- mice. High p-Akt activity (nuclear and cytoplasmic) in the dorsal prostate of Id4-/- mice was consistent with decreased Pten expression (Figure 4D). Unexpectedly, low to negligible p-Akt activity was observed in the ventral (Figure 4E) and lateral prostates (Figure 4F) suggesting a lobe specific effect. We reasoned that decreased p-Akt even in the absence of Pten could be due to reduced expression of total Akt. Surprisingly, total Akt expression was undetectable in lateral and ventral prostate (Figure 4G) but was present in dorsal prostate (Figure 4H and I). These results suggested that loss of p-AKt observed in lateral and ventral prostate was likely due to decreased expression of total Akt and not due to loss of Pten. High Akt expression was observed in the wild type prostate but the expression pattern was unanticipated. Akt expression in the glandular epithelium was not uniform but highly localized to few cells (Figure 4J-L) suggesting that Akt expression is not constitutive. The expression of p-Akt was consistent with regions expressing high and low Akt. We next counted p-Akt positive cells in tubules that also stained positive for Akt (see panels D, H and I in Figure 4). A significant (P < 0.001) increase in the fraction of p-Akt positive cells in this analysis (inset in Figure 4L) further supports the lack of Pten in Id4-/- prostates as compared to Id4+/+ prostate. Additional studies will be required to demonstrate the functional significance of the localized Akt expression and its effect on cell function, for example whether Akt expression correlates with pAKt, Pten and downstream effectors of p-Akt in specific Akt positive and negative cell types in a lobe specific manner.

#### Id4 and Proliferative defect: Loss of Id4 promotes proliferation without altering apoptosis

**Figure 5:**
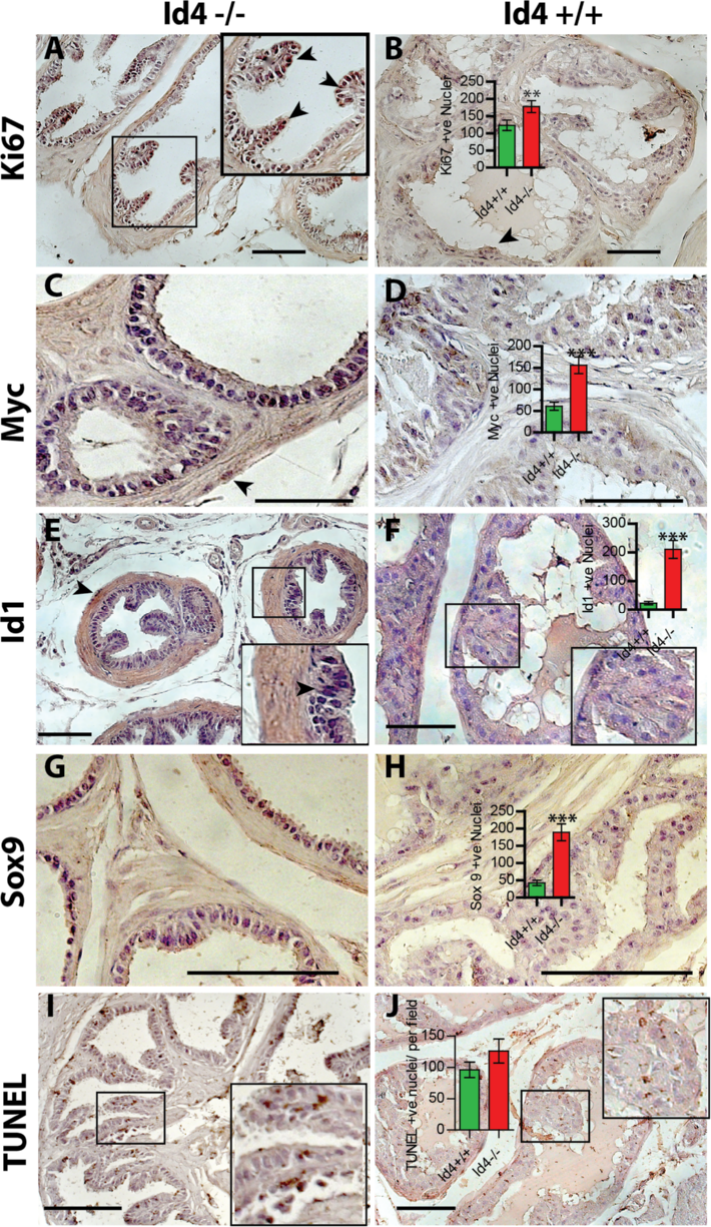
**Id4 expression is associated with proliferation (Ki67), apoptosis (TUNEL) and proliferative markers (Myc, Id1 and Sox9). A:** Increased proliferation represented by high Ki67 immuno-staining was observed in Id4-/-. Cells at the tips of the finger like projections stained more strongly with Ki67 as compared to those near the basement membrane (inset in Panel **A**). Panel **B**: High Ki67 was observed only in a few cells in the glandular epithelial cells of the wild type (Id4+/+) prostate (black arrowheads). Panels **C**- **F**: High nuclear Myc (**C**) and Id1 (**E**, and inset) expression was observed in the Id4-/- epithelial cells as compared to wild type littermates (**D** and **F** respectively). Panels **G** and **H:** Nuclear Sox9 expression was absent in the wild type (Id4+/+, Panel **H**) but high nuclear Sox9 expression was observed in the Id4-/- glandular epithelial cells of the prostate (Panel **G**). Panels **I** and **J**: TUNEL assay demonstrated increased apoptosis in in Id4 -/- mice prostates (Panel **I** and inset, Brown staining) as compared to the wild type littermates (Panel **D** and inset). The graph in panel **J** represents average number of TUNEL positive cells per field. The TUNEL positive cells were counted in five fields (at 400×) on three different tissue samples. Average number of TUNEL positive cells per field represented as mean + SEM in Id4-/- were not statistically different from WT littermates. Representative images are shown. The scale bar is 100um. The respective Ki67 (**B**), Myc (**D**), Id1 (**F**), Sox9 (**H**) and Tunel (**J**) positive cells were counted (n = 25 tubules) in Id4+/+ and Id4-/- cross sections and subjected to *t*-test (** P < 0.01, *** P < 0.001, inset).

The presence of hyperplastic regions (Figure 2O-R) was associated with increased expression of proliferative marker Ki67 in Id4-/- in prostate ducts (Figure 5A). Marked increase in Ki67 was also observed in growing prostatic projections in the lumen in Id4-/- prostates (Box in Figure 5A). In contrast, Ki67 positive nuclei in Id4+/+ littermates were observed in only few cells per tubule (Figure 5B, P < 0.01, Ki67 positive nuclei counted in n = 25 tubules).

Increased Ki67 was also associated with increased expression of regulators of proliferation such as Myc [44] and Id1 [45]. Myc positive nuclei were more frequently observed in glandular epithelial cells in Id4-/- (Figure 5C) as compared to Id4+/+ prostates (Figure 5D, P < 0.001, Myc positive nuclei counted in n = 25 tubules). Recent studies have also shown an inverse relationship between Myc and Nkx3.1 [46,47].

Id1, a member of the HLH family of transcription factors was undetectable in the Id4+/+ (Figure 5F) but increased significantly in Id4-/- mice (Figure 5E, P < 0.001, Id1 positive nuclei counted in n = 25 tubules). Id1 promotes cell cycle progression by down-regulating multiple CDKNIs including p21 and p16(Ink4a) [48,49]. Together with Myc, increased Id1 expression is also associated with increasing grade of prostate cancer [33,34,50].

In Pten and Nkx3.1 mutant mice, cells with increased levels of SOX9 are persistently present within prostate epithelia [51]. Immuno-histochemical analysis using Sox9 antibody revealed few Sox9 positive luminal epithelial cells in the wild type prostates (Figure 5H) [52]. In contrast, the epithelial cells from the Id4-/- prostate showed significantly higher Sox9 expression (Figure 5G, P < 0.001, Sox9 positive nuclei counted in n = 25 tubules). Increased Sox9 expression is observed at early stages of prostate hyperplasia and is associated with progression to high grade PIN lesions [53]. Sox9 is part of the prostate developmental pathway that is reactivated in prostate neoplasia where it promotes tumor cell proliferation and correlates with Ki67 expression [51].

The average number of TUNEL positive cells (from 5 different fields) in Id4-/- mice prostate (Figure 5I and inset) was not significantly different from WT mice (Figure 5J and inset, and graph representing TUNEL positive nuclei counted in n = 25 tubules). We speculate that even a small increase in proliferation, without noticeable change in apoptosis could have a dramatic effect on cellular growth.

**Figure 6:**
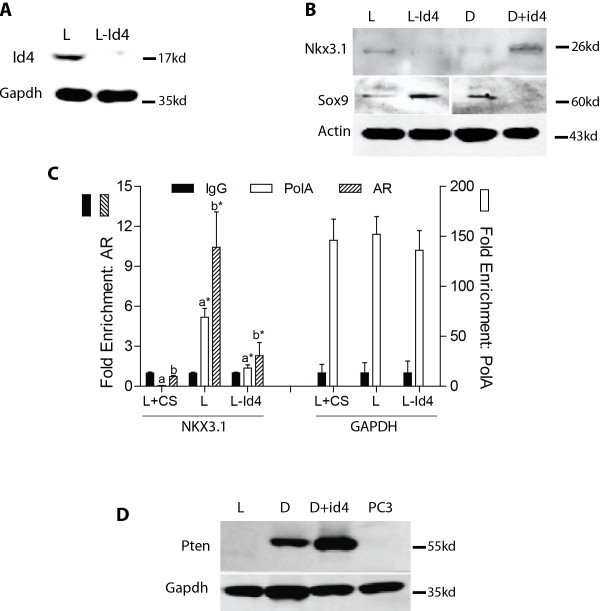
**Id4 expression in prostate cancer cell lines LNCaP and DU145 is associated with NKX3.1, Sox9 and PTEN expression.** Panel **A**: Id4 was silenced in LNCaP (L) cells with gene specific shRNA (L-Id4). Panel **B:** NKX3.1 and Sox9 expression in LNCaP cells in which Id4 was silenced (Panel **A**) or in DU45 cells (**D**) in which Id4 was ectopically expressed (D + Id4). Panel **C**: Chromatin immuno-precipitation to demonstrate occupancy of androgen receptor (AR) at the androgen response element on NKX3.1 promoter in LNCaP (L) and LNCaP-Id4 (L-Id4) cells. Polymerase A (PolA) enrichment on GAPDH promoter was used as an internal control. The data represented as mean ± SEM of three different experiments is normalized IgG (CS: Charcoal stripped FBS, a* and b*: p < 0.001 as compared to a and b respectively). Note that PolA enrichment is shown on the right y-axis and IgG and ARE on the left y-axis. Panel **D:** PTEN protein is undetectable in LNCaP cells due to frame shift mutation. Increased PTEN expression is observed in DU145 cells in which Id4 is constitutively expressed (D + Id4). PTEN null prostate cancer cell line PC3 was used as a negative control for PTEN expression. Representative of three different experiments is shown.

The molecular changes in the prostate observed in the Id4-/- mouse model were further confirmed *in vitro* using Id4 gene silencing and gain-of-function approaches in the prostate cancer cell lines LNCaP and DU145. Id4 was silenced in LNCaP cells using gene specific siRNA (Figure 6A) and over-expressed in DU145 cells as previously described [29,35]. Similar to the Id4-/- studies as described above (Figure 3D and F), silencing of Id4 in LNCaP cells resulted in decreased NKX3.1 expression, whereas ectopic Id4 expression in DU145 increased NKX3.1 expression (Figure 6B). Consistent with lack of androgen receptor binding on NKX3.1 promoter in Id4-/- mice prostate (Figure 3H), a significant decrease (P < 0.001) in androgen receptor binding on consensus ARE in NKX3.1 promoter (-3013 bp relative to transcriptional start site) was observed in LNCaP-Id4 cells as compared LNCaP cells (Figure 6C). These results clearly demonstrated that NKX3.1 is dependent on Id4. Loss of Id4 in LNCaP cells also resulted in increased Sox9 in these cells whereas Sox9 was undetectable in DU145 + Id4 cells (Figure 6B). Due to frame shift mutation, PTEN protein expression is not observed in LNCaP cells (Figure 6D) [54]. However, PTEN expression was higher in DU145 + Id4 cells as compared to DU145 cells alone (Figure 6D). These results not only confirmed the molecular changes observed in our in vivo and in vitro models but strongly support the role of Id4 as a potential tumor suppressor that is required for normal prostate development also.

## Discussion

This study supports a role for Id4 as a key regulator of male genital tract development. Although we focused on the prostate, the size and development of accessory sex glands (seminal vesicles) and testis is also severely impaired. Id4 may not be required to maintain fertility [15] but it could cooperate with other possibly overlapping regulatory genes to support normal development of various organs within the genital tract.

Genital tract development in general and prostate in particular are androgen dependent. Prostate fetal development, structural and functional maturation at puberty is strictly androgen regulated [55]. Loss of androgen receptor, specifically in the prostate epithelial cells (PEARKO, prostate epithelial AR knockout) leads to a phenotype [56,57] that is very similar to the Id4-/- prostates e.g. increased proliferation, decreased size and number of tubules and lack of differentiated epithelial cells. Based on the chromatin immuno-precipitation studies of the mouse Nkx3.1 promoter and increased NKX.3.1 expression in DU145 + Id4 cells, we propose that Id4 is required to maintain certain facets of androgen receptor activity in the prostate epithelium. In particular, Id4 could support the function of the AR as a suppressor of epithelial proliferation in the mature prostate, which is defective in prostate cancer [58].

*Nkx3.1* regulates early postnatal ductal morphogenesis and maintains normal differentiation of the prostate epithelium including the production of secretory proteins [38,39]. Similar to Nkx3.1-/- mice, the Id4-/- mice also display reduced ductal branching morphogenesis, epithelial hyperplasia and dysplasia. But unlike Id4-/- mice, the overall prostate sizes and wet weights in *Nkx3.1 -/-* and +/+ mice [39] are similar. Nevertheless, loss of Nkx3.1, a marker of epithelial differentiation and androgen response is a significant observation that further supports the attenuation of androgen regulatory network post androgen receptor expression in the Id4-/- prostates.

Nkx3.1 also regulates the rate at which proliferating luminal epithelial cells exit the cell cycle and its loss extends the transient proliferative phase of luminal cells [59] which is consistent with increased expression of ki67, Myc and Id1 in Id4-/- prostate. An increase in the Myc:Nkx3.1 ratio observed in Id4-/- mice could also promote Myc dependent transactivation of pro-tumorigenic target genes [47]. Conversely, a decrease in Myc:Nkx3.1 ratio may promote Nkx3.1 dependent transactivation of anti-tumorigenic target genes. Mice expressing Myc in the prostate also develop PIN like lesions followed by invasive adenocarcinoma [60]. Inactivation of Pten also promotes cellular Myc activation [42] which is consistent with our results. Thus, some of the phenotypes resulting from the loss of Nkx3.1 are consistent with the literature but the smaller prostate size in Id4-/- mice appears to result also from alterations of other regulatory pathways that could be independent of Nkx3.1 such as Akt signaling (see below).

Id1 is also a member of helix-loop-helix family of transcriptional regulators that contributes to cell proliferation and restrains differentiation and apoptosis [61,62]. Both Id1 and Id4 share strong sequence homology and interact with similar bHLH proteins for example TCF3, but their expression patterns are largely non-overlapping [61]. We and others have shown that Id4 and Id1 expression is mutually exclusive in the normal prostate [35] and prostate cancer [29-31,33,50,63]. Such a mutually exclusive expression pattern is also observed in the Id4-/- mice further suggesting loss of epithelial differentiation and increased proliferation. Sustained Id1 expression also failed to rescue the Id4-/- deficient phenotype supporting the argument that these two structurally similar proteins are functionally divergent and non-compensatory.

Sox9 is critical for maintaining the basal epithelial cells in tissues and may have a similar function in prostate epithelium [64]. In the adult prostate, SOX9 is expressed diffusely in the basal cell layer suggesting that it is required for maintaining basal cell function. These basal cells represent and/or include prostate stem cells also [65]. Increased Sox9 expression in the prostate epithelial component may suggest the expansion of this basal cell population that remains undifferentiated as evidenced by persistent Id1 expression, increased proliferation (lack of exit from cell cycle) and decreased differentiation markers (Nkx3.1). However direct studies identifying specific basal cell populations (e.g. p63 expression) and/or stem cell markers and there transitions to specific cell types will be required to further consolidate this specific mechanism.

Investigating whether loss of Id4 results in an early defect or is a later post-pubertal effect will be required to fully comprehend the scope of Id4 in the regulation of prostate development. Whether Id4 is vital to maintain a specific Sox9 positive prostate stem cell component that eventually expands to promote normal prostate development is an interesting proposition based on two different studies. First, Id4 is required for neuronal stem cell maintenance but a relatively mild mutant phenotype is observed at post natal day 0 despite the early loss of stem cells due to both premature differentiation and compromised cell cycle transition [13]. Second, in mice lacking Id4 expression, quantitatively normal spermatogenesis is impaired due to progressive loss of the undifferentiated spermatogonial stem cell population during adulthood [15]. These studies indicated that Id4 is a distinguishing marker of spermatogonial stem cells in the mammalian germline and plays an important role in the regulation of self-renewal. The observations made in the later study are particularly exciting given the overall impact of Id4-/- on the male reproductive tract and suggests a potential common molecular mechanism of action targeting a stem cell population in various organs of the male reproductive tract. In the prostate, Id4 could also be expressed in a specific stem cell population such as Sca-1^hi^, Sca-1^lo^, Sca-negative [66] and/or their progenitors that contribute to the prostate phenotype in Id4-/- mice.

Loss of Id4-/- also impairs mammary gland development [14]. In the mammary gland, Id4 expression is mainly observed in the cap cells, basal cells and in a subset of luminal cells, whereas in the prostate Id4, expression is primarily in the luminal epithelial cells. Conceptually, reduced ductal branching in prostate is similar to reduced ductal branching/expansion and branching morphogenesis in mammary gland of Id4-/- mice. In mammary gland, loss of Id4-/- is associated with reduced cellular proliferation but in the prostate, loss of Id4 was associated with increased proliferation (Ki67) and decreased differentiation (Nkx3.1) suggesting that the regulatory role of Id4 in mammary gland and prostate are distinct.

The presence of focal hyperplastic regions resembling PIN like lesions is also observed in Id4-/- mice. Many of the genes associated with prostate cancer and their respective knockout/transgenic phenotypes are also recapitulated in the Id4-/- model that support the role of Id4 in prostate cancer. Apart from loss of Nkx3.1 as discussed above, a decrease in Pten specifically in the prostate, sustained androgen receptor expression, increased Myc and Sox9 also promote early stages prostatic intraepithelial neoplasia [51]. Our results suggest that the above noted genes and their regulated pathways are downstream of Id4. However, in spite of these complex alterations, we did not observe a significantly greater number of pre-neoplastic lesions in Id4-/- prostate suggesting the possibility of mechanisms/pathways that restrains the formation of significant pre-cancerous lesions and prostate cancer. One of these pathways could involve Akt, a kinase on which many of these pathways converge. Akt1 and 2 deficiency is sufficient to markedly reduce the incidence of tumors in Pten(+/-) mice [67] and Myc also cooperates with Akt1 in promoting prostate tumorigenesis [68]. Thus loss of Akt could be a key mechanism that negatively regulates the formation of PIN like lesions given the remarkable pro-neoplastic gene signature in Id4-/- mice. Loss of Akt1 also leads to increased apoptosis and general growth retardation that affect the size of organs [69]. We speculate that the smaller genital tract and prostate in Id4-/- could be in part due to decreased Akt expression.

Based on sequence homology and interaction studies, Id4 could still function as a dominant negative inhibitor of bHLH transcription factor of the E2A (TCF3) family. However, its interactions with non-bHLH proteins could be the key to understand it’s pro-differentiation vs. inhibitor of differentiation functions. For example, in response to BMP4, Id4 stabilizes RUNX2 and promotes osteoblast differentiation [16]. A similar mechanism can be envisioned in the prostate where Id4 could stabilize transcription factors involved in prostate development such as the Homeobox (*Hox* cluster, and Nkx3.1) and Forkhead box genes (Fox A1 and A2) in response to secreted signaling molecules (*Wnts, Fgfs, BMPs/TGFβ/Activins*) [70]. These complex interactions and cross-regulation could promote Id4 dependent prostate morphoregulatory gene signature essential for normal prostate development. Id4 could also regulate the correct timing of prostate epithelial cell differentiation, in a mechanism similar to neural differentiation [71] through complex interplay involving transcription factors (bHLH and non-bHLH) and response to signals from the surrounding mesenchyme.

## Conclusions

**Figure 7:**
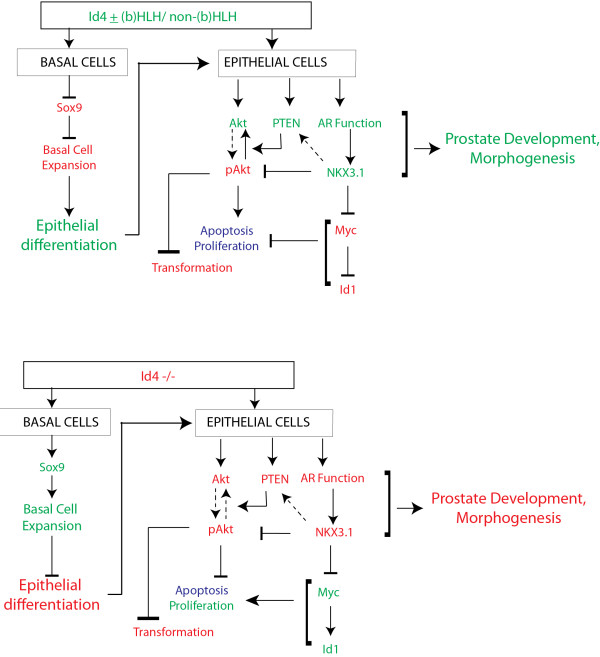
**Proposed mechanism of action of Id4 in prostate development.** The letters in green and red indicate induction/stimulation or repression/restrain of a gene/process respectively. The letters in blue indicate no change or balance for example a balance is maintained between apoptosis and proliferation. The schematic incorporates data from this study and published studies that are referenced in the text.

The Id4-/- knockout presents a complex prostate phenotype. Loss of Id4 results in altered prostate development but also leads to or promotes some PIN like lesions that are supported both by morphological and specific marker studies. At least three potential Id4-/- dependent mechanisms can be conceptualized (Figure 7): First, the altered androgen-receptor – Id4 interaction pathway in which Id4 is required to promote androgen dependent differentiation program. This mechanism is supported by the Id4 dependent Nkx3.1 expression as shown in normal prostate epithelial cells, Chromatin immuno-precipitation studies, androgen sensitive prostate cancer cell lines and similarities of the prostate phenotype with PEARKO mice. Second, a stem cell hypothesis wherein Id4 is required to maintain or influence the timing of differentiation of a specific stem cell population, and third, basal cell expansion in which epithelial differentiation is blocked due to persistent Sox9 expression. Alteration in any of these pathways could result in abnormal prostate and reproductive tract development and may establish gene expression signatures that favor (PTEN, NKX3.1, Id1, Myc) or restrain (Akt) development of prostate gland and pre-cancerous lesions.

## Materials and methods

### Animals

All animal studies were conducted in accordance with federal guidelines and approved by the Institutional Animal Care and Use Committee, Geisel Medical School at Dartmouth. The mice were sedated using a lethal dose of tribromoethanol (TBE) followed by terminal perfusion with 10% acetate buffered formalin. The reproductive tract including prostates from 6-8 weeks old Id4-/-, Id4+/- and Id4+/+ mice were obtained from Dr. Mark A. Israel (Norris Cotton Cancer Center, Lebanon, NH, USA). The Id4-/- mice were generated by targeted replacement of the endogenous Id4 locus with the green fluorescent protein (GFP) coding sequence [13]. The tissues were fixed in buffered formalin and paraffin embedded.

### Histological analysis

Five micron sections were used for all histological and immuno-histochemical analysis. The sections were stained with hematoxylin and eosin using standard procedures. The H&E sections from knockout, heterozygous and wild type mice were examined by veterinary pathologists (Dr. Thomas Graham, DVM, PhD, and Dr. Ebony Gilbreath, DVM, PhD, Department of Pathobiology, School of Veterinary Medicine, Tuskegee University, Tuskegee, AL, USA). All the sections were performed from proximal to distal region with ventral prostate as the most proximal region.

### Immuno-histological analysis

Slides were processed through standard protocols. Following antigen retrieval (autoclave in 0.01 M sodium citrate buffer pH 6.0 at 121C/20 psi for 30 min), the peroxidase activity was blocked in 3% H_2_O_2_ and non-specific binding sites blocked in 10% Goat serum. The blocked sections were incubated overnight at 4°C with either of the following antibodies: Androgen receptor (Rabbit mAb, Cell Signaling, cat#153P), Akt (11E7, Rabbit mAB, Cell Signaling, cat# 4685), phospho Akt (ser473, Rabbit pAb, Cell Signaling, cat# 9271), Pten (Rabbit mAb, Cell Signaling, cat#9559), Myc (Rabbit mAb, Cell Signaling, cat#5605X), NKX3.1 (mouse mAb, Thermo Scientific, cat#16906), Sox9 (Rabbit pAb, Novus biological, NB-100-2202), Id4 (Rabbit pAb, Aviva, ARP38058-T100), Id1 (Rabbit mAb, cat# BCH-1#195-14), Ki67 (Rabbit polyclonal, AbCam, #ab15580) followed by incubation with secondary antibody (goat anti-rabbit (#32260) or goat anti-mouse (#32230) -HRP, Thermo Scientific) for 1 hour. The slides were stained with DAB for 2 min, counterstained with hematoxylin and mounted with Immuno-mount (Thermo Scientific), examined and photo-micrographs taken using the Zeiss microscope with an AxioVision version 4.8 imaging system. All the antibodies were mono-reactive, that is a single reactive band was observed in western blot using total cell lysate from prostate cancer cell lines LNCaP, DU1545 and PC3. Non-specific binding of the secondary antibodies was evaluated using respective normal IgGs (data not shown).

### TUNEL assay

The terminal deoxynucleotidyl transferase-mediated deoxyuridine triphosphate (dUTP) nick end labeling (TUNEL) assay was used to detect fragmented DNA as marker for apoptosis in FFPE tissue sections using TACS 2 TdT-DAB apoptosis detection kit (Trevigen). The slides were counterstained in hematoxylin and mounted with Immuno-mount (Thermo Scientific).

### Id4 over-expression and silencing in prostate cancer cell lines

The prostate cancer cell lines LNCaP, DU145 and PC3 were purchased from ATCC and cultured as per ATCC recommendations. Human Id4 was over-expressed in DU145 cells as previously described [35]. Id4 was stably silenced in LNCaP cells using a gene specific shRNA retroviral vector (Open Biosystems #RHS1764-97196818). Successful Id4 over-expression and gene silencing was confirmed by qRT-PCR and Western blot analysis.

### Western blot analysis

Total cellular protein was prepared from cultured prostate cancer cell lines using M-PER (Thermo Scientific). 30ug of total protein was size fractionated on 4-20% SDS-polyacrylamide gel (Novex) and subsequently blotted onto a nitrocellulose membrane (Whatman). The blotted nitrocellulose membrane was subjected to western blot analysis using protein specific antibodies as mentioned above. After washing with 1x PBS with 0.5% Tween 20, the membranes were incubated with a horseradish peroxidase (*HRP*) coupled secondary antibody against rabbit or mouse IgG and visualized using the Super Signal West Dura Extended Duration Substrate (Thermo Scientific) on Fuji Film LAS-3000 Imager.

### Chromatin immuno-precipitation (ChIP) assay

Formalin-fixed paraffin-embedded (FFPE) samples from wild type and Id4 knockout mice were used for ChIP based analysis of androgen receptor binding on the mouse Nkx3.1 promoter. For this analysis, 40 μm thick FFPE sections with more that 75% prostatic ducts were used from Id4-/- and WT mice. Genomic DNA was isolated from these sections by the method of Fanelli et al., [72] except that tissue samples were de-paraffinized with xylene instead of histolemon. The chromatin extracted from tissue samples was sheared (Covaris S220), subjected to immuno-precipitation with either androgen receptor (Millipore, #06-680), mouse IgG (Active motif # 102302) or RNA polI (Millipore, #05-623) antibodies, reverse cross linked and subjected to qRT- PCR [72]. The androgen receptor binding site (AAA TTA TGG ATG TTC TTT TAA GTC TT) in the first intron of mouse Nkx3.1 [40] (311 bp from start of first intron) was quantitated by real time PCR (BioRad CFX96) using forward (5′GCC CAC TCT TAA GTT CCC TT) and reverse (5′CAT GAA AAG TGG TTG GGG CC) primers (130 bp amplicon).

LNCaP and LNCaP-Id4 cells cultured in 10% Fetal bovine serum were used to analyze androgen receptor binding on consensus ARE sites in NKX3.1 promoter using primer pairs described previously [73] with EZ CHiP kit (Millipore). The reagents for PolA CHiP on GAPDH were included in the EZ CHiP kit as internal standards.

### Data and statistical analysis

The NIH Image J [74] was used for counting, calculation of area and diameter of H&E stained prostatic ducts (for description see respective figure legends). Quantitative real time data was analyzed using the ∆∆Ct method: the Ct values of IgG were used to first calculate ∆Ct. Following this normalization step, the ∆∆Ct was then calculated with ∆Ct of wild type set to 1. Within group Student’s *t*-test was used for evaluating the statistical differences between groups. One-way ANOVA and Dunnett’s multiple tests were used to test for differences between more than two groups.

## Competing interests

The authors declare that they have no competing interests.

## Authors’ contributions

PS: Immuno-histochemistry, data analysis and first draft of manuscript. AEK: Development of LNCaP-Id4 cell lines. SC: Chomatin immuno-precipitation assays. SK: Western blot analysis. PN: Histology. DP: Prepared samples for Chromatin immuno-precipitations. MCH: Maintenance of Id4 KO mice and dissection of prostates. JC: Conceived the study and final draft of the manuscript. All authors read and approved the final manuscript.
